# Discovery of Infection Associated Metabolic Markers in Human African Trypanosomiasis

**DOI:** 10.1371/journal.pntd.0004200

**Published:** 2015-10-27

**Authors:** Sabrina D. Lamour, Maria Gomez-Romero, Panagiotis A. Vorkas, Vincent P. Alibu, Jasmina Saric, Elaine Holmes, Jeremy M. Sternberg

**Affiliations:** 1 Section of Biomolecular Medicine, Division of Computational and Systems Medicine, Department of Surgery and Cancer, Imperial College London, London, United Kingdom; 2 Section of Hepatology and Gastroenterology, Department of Medicine, Imperial College London, London, United Kingdom; 3 Institute of Biological and Environmental Sciences, University Of Aberdeen, Aberdeen, United Kingdom; Institute of Tropical Medicine, BELGIUM

## Abstract

Human African trypanosomiasis (HAT) remains a major neglected tropical disease in Sub-Saharan Africa. As clinical symptoms are usually non-specific, new diagnostic and prognostic markers are urgently needed to enhance the number of identified cases and optimise treatment. This is particularly important for disease caused by *Trypanosoma brucei rhodesiense*, where indirect immunodiagnostic approaches have to date been unsuccessful. We have conducted global metabolic profiling of plasma from *T*.*b*.*rhodesiense* HAT patients and endemic controls, using ^1^H nuclear magnetic resonance (NMR) spectroscopy and ultra-performance liquid chromatography, coupled with mass spectrometry (UPLC-MS) and identified differences in the lipid, amino acid and metabolite profiles. Altogether 16 significantly disease discriminatory metabolite markers were found using NMR, and a further 37 lipid markers via UPLC-MS. These included significantly higher levels of phenylalanine, formate, creatinine, *N*-acetylated glycoprotein and triglycerides in patients relative to controls. HAT patients also displayed lower concentrations of histidine, sphingomyelins, lysophosphatidylcholines, and several polyunsaturated phosphatidylcholines. While the disease metabolite profile was partially consistent with previous data published in experimental rodent infection, we also found unique lipid and amino acid profile markers highlighting subtle but important differences between the host response to trypanosome infections between animal models and natural human infections. Our results demonstrate the potential of metabolic profiling in the identification of novel diagnostic biomarkers and the elucidation of pathogenetic mechanisms in this disease.

## Author Summary

Metabolic profiling of biofluids and tissues in disease and healthy individuals is a powerful approach to discover new markers for diagnosis. We have applied these techniques to the protozoan infection human African trypanosomiasis (HAT), otherwise known as sleeping sickness. The form of HAT endemic in East Africa, caused by *Trypanosoma brucei rhodesiense*, requires technically demanding direct microscopic diagnosis, as there are no indirect rapid diagnostic tests available. We studied the metabolite profiles in plasma from HAT patients and controls. Clear biochemical differences were discovered between control individuals and patients, including changes in the overall lipid composition and concentration of certain amino acids. These may have been caused by the inflammatory immune response to infection and the uptake of particular molecules by the parasites, although further research will be required for confirmation. We demonstrate that plasma metabolic profiles are characteristic for *T*. *b*. *rhodesiense* infection. While some of these changes are consistent with those observed in an experimental mouse infection model of HAT, many are unique to this clinical study and indicate the necessity of validating experimental animal study data in clinical disease studies. Our results also reveal biochemical changes in patients that will help us understand the development of disease.

## Introduction

Human African Trypanosomiasis (HAT) is caused by infection with either of two subspecies of *Trypanosoma brucei*. *Trypanosoma brucei (T*. *b*.*) gambiense* causes chronic disease (that can last months or years) and is endemic in Western Africa while *T*. *b*. *rhodiesiense* causes a more acute illness in Eastern and Southern Africa, which is typically fatal within less than a year of infection if untreated [1,2]. Diagnosis is a critical challenge for treatment and control of this disease as patients display an array of non-specific inflammatory symptoms, often indistinguishable from other endemic illnesses such as malaria or enteric fever [1,3]. Infection with *T*. *b*. *gambiense* is routinely screened using the Card Agglutination Test for Trypanosomiasis (CATT) and also a new generation of lateral-flow rapid diagnostic tests are being deployed based on the host-response to commonly expressed variant surface glycoproteins (VSG) [4]. However no such serological approach has been successful for *T*. *b*. *rhodesiense* probably due to the greater diversity of VSG expression. New diagnostic techniques are urgently needed for this disease in order to meet the World Health Organization (WHO) target of elimination by 2020 [5,6].

In search for novel markers of disease, we have conducted global untargeted metabolic profiling of plasma from *rhodesiense* trypanosomiasis patients from Uganda and matched controls. Metabolic profiling (also termed metabonomics/metabolomics) can be used to characterise biochemical patterns associated with specific physiological and/or pathological states [7]. In the field of parasitic infections, this approach has shown capacity for characterising infection-induced metabolic changes in the host, within infected tissues and systemically (as measured by plasma or urine profiles). As yet, parasitic metabolic profiling studies have largely focused on *in vitro* assays [8–10] and experimental rodent models [11–15]. There have been very few examples of identifying the metabolic signature of a specific parasitic infection in humans [16], largely because human profiles are confounded by strong genetic and environmental variation, and are often superimposed upon a background of other concurrent endemic infections. In mice, infection with *T*. *b*. *brucei* (a subspecies of *T*. *brucei* that is not infective to humans) resulted in augmented plasma levels of lactate, acetylglycoproteins and creatine, and reductions in phosphatidylcholine and lipoproteins, as well as elevated pro-inflammatory cytokines [11,17]. Increases in plasma pro-inflammatory cytokines have also been measured in clinical samples [18,19], although metabolic similarities between the mouse model and humans remain to be investigated. In this study we present the first characterisation the metabolic effects of *T*. *brucei rhodesiense* infection in humans, and highlight both similarities and differences to results from published mouse model infections with *T*.*brucei brucei*.

Plasma samples were analysed by proton nuclear magnetic resonance (^1^H NMR) spectroscopy and reversed-phase ultra-performance liquid chromatography, coupled to mass spectrometry (RP-UPLC-MS). ^1^H NMR is a highly reproducible method optimal for analysing complex biological mixtures, such as plasma, with minimal sample preparation [20]. Evidence from a range of experimental models have highlighted marked changes in plasma lipids following *T*. *brucei ssp* infection, such as hypertriglyceridaemia [11,21–23]. Consequently, lipid profiles were separately characterised via UPLC-MS, to provide complementary information on the different lipid species altered in disease. We hypothesised that the metabolic phenotype of individuals with HAT *rhodesiense* infection would be distinct from that of uninfected individuals, yielding a range of discriminatory markers that may be of diagnostic value.

## Methods

### Ethics Statement

The human plasma samples in this study were collected under protocols approved by ethics committees in Uganda (UNCST) and UK (North of Scotland Research Ethics Committee), conforming to the principles of the Declaration of Helsinki. Ethical consent forms and information sheets were designed in English and translated into local languages. Informed consent was given as a signature or a thumb-print (as approved by the UNCST) after verbal explanation. For those aged under 18, affirmative consent as well as the consent of their legal guardian was obtained.

### Patient Information and Sample Collection

A total of 46 HAT patients and 21 controls were recruited at Lwala Hospital, Kaberamaido District and Serere Health Centre, Serere District in Eastern Uganda, between November 2008 and March 2010. Controls were healthy individuals who were confirmed as non-infected with either trypanosomes or *Plasmodium sp*. having undergone parasitological assessment. Study sites, recruitment protocols, treatment regimens, disease progression characteristics and clinical examination methods have been published elsewhere [24]. Patients with intercurrent infections of malaria, filariasis or schistosomiasis were excluded. Plasma and cerebral spinal fluid (CSF) samples were collected from all patients prior to treatment as part of normal diagnostic and staging procedures. Staging was carried out in accordance with WHO criteria [25] defining late stage by the presence of parasites in the lumbar CSF and/or a CSF white blood cell count (WBC) > 5/μl. Aliquots of 1–2 ml of plasma were immediately frozen and then maintained in liquid nitrogen until transfer to the UK. After air-freight (24 hours on dry ice) samples were maintained at -80°C until analysis. A summary of the parasitological and demographic characteristics of the patients is presented in supplementary S1 Table.

### ^1^H NMR Acquisition and Processing

All sample preparation was performed in one batch in a randomised order. Prior to acquisition, plasma samples were diluted 1:1 with plasma buffer (0.142 M NaHPO_4_, 2 mM NaN_3_, 0.08% (volume/volume i.e. v/v) 3-(Trimethylsilyl)propionic-2,2,3,3-d_4_ acid sodium salt (TSP) solution, 20% (v/v) D_2_O; all SIGMA-Aldrich, Germany), and transferred into 5 mm NMR tubes. ^1^H NMR data were acquired (over 3 days) on a Bruker Avance 600 MHz (14.1 T) NMR Spectrometer with BB probe head and refrigerated SampleJet autosampler (Bruker, Germany), using Topsin (3.1) software (Bruker BioSpin, Germany). Samples were acquired at 300 K (~27°C) using a standard one-dimensional pulse with water suppression program for general metabolite screening [26]; sequence as follows: 2 second (s) relaxation delay (RD)– 90° pulse– 4 μs delay—90° pulse– 100 μs mixing time– 90° pulse– 2.73 s acquisition (ACQ) of free induction decay (FID) signal. Number of dummy scans (ds) was 4, and number of acquired FID’s (ns) was 64. To enhance the visualisation of low molecular weight molecules, a Carr-Purcell Meiboom-Gill (CPMG) spin-echo pulse spectrum was also acquired for each sample [26], with the following pulse sequence: 2 s RD– 90° pulse–(τ– 180°–τ)_n_-ACQ, where spin echo time τ = 300 μs, number of loops n = 128, total spin echo time 2nτ = 76.8 ms, ds = 4 and ns = 64. All FID’s were multiplied by an exponential function with a 0.3 Hz line broadening prior to Fourier transformation into spectral data (spectral width of 18 kHz collected over 131,072 data points). Spectra were processed using automatic phasing, baseline correction and calibrated to the TSP peak via an in-house algorithm in MATLAB (version R2013a, Mathworks Inc.) and Topspin. The residual water signal was removed prior to automatic spectral alignment [27], and probabilistic quotient normalisation [28], using an in-house MATLAB script. Additionally, artefactual peaks arising from EDTA buffer [29] present in blood collection tubes were removed from all plasma spectra for final analysis. The regions removed were 2.53–2.62, 2.7–2.735, 3.08–3.256, 3.60–3.66 ppm which may have resulted in the loss of some endogenous metabolic information. Spectral assignments of metabolite peaks were identified using Chenomx NMR suite profiler 8.1 software and known assignments from in-house NMR databases and literature [30,31].

### Plasma Lipid Profiling UPLC-MS Acquisition and Processing

Due to sample limitations, additional aliquots of only a subset of plasma samples were used for lipid profiling (*n* = 30, see supplementary S1 Table). As for NMR, sample preparation for UPLC-MS was performed in one day in one batch, in a randomised manner. Samples underwent protein precipitation using isopropanol, as described by Sarafian et al, 2014 [32]. Briefly, 50 μl plasma was mixed 1:3 (v/v) with ice-cold isopropanol (SIGMA-Aldrich), incubated for 10 mins at room temperature, and then overnight at -20°C. Samples were then centrifuged at 20,817 x g (14,000 rpm) at 4°C for 20 mins and 100 μl of the supernatant (subsequently referred to as test samples) were transferred to MS vials (Waters Corp., UK) for analysis and placed in the auto-sampler (kept at 8°C). Additional aliquots from each plasma-isopropanol sample were pooled to generate a quality control (QC) master sample. The QC was injected before starting the run to optimise the injection volume and to condition the column, and then injected every 8 samples for assurance of a stable run.

Lipid profiling was performed on an Acquity UPLC system (Waters Ltd. Elstree, UK) coupled to a Q-TOF Premier mass spectrometer (Waters Ltd., Manchester, UK). Mass spectra for all samples were acquired continuously, firstly in positive and then negative ion electrospray (ESI+ and ESI-) modes, over the course of 3 days. The separation conditions for reversed-phase UPLC have been previously established [33,34]. Gradient elution was performed using a CSH C_18_ (1.7 μm, 2.1 x 100 mm) column (Waters Corporation, Milford, USA) kept at 55°C. The mobile phases consisted of 0.1% (v/v) formic acid and 10 mM ammonium formate in 60:40 (v/v) acetonitrile (ACN)/HPLC-grade water (mobile phase A) and 0.1% formic acid (v/v) and 10 mM ammonium formate in 90:10 (v/v) IPA/ACN (mobile phase B) at a flow rate of 0.4 ml/min (all solvents purchased from SIGMA-Aldrich except water which was from Fisher, Germany). The sample injection volume was 5 μl and 10 μl for ESI+ and ESI-, respectively.

The MS parameters were set as follows: capillary voltage, 3 kV (ESI+) and 2.5 kV (ESI-); sample cone voltage, 30 V (ESI+) and 25 V (ESI-); source temperature, 120°C; desolvation temperature, 400°C; desolvation gas flow, 800 L/h and cone gas flow, 25 L/h. For mass accuracy, a 0.2 ng/μl leucine encephalin (mass/charge ratio (m/z) 556.2771 in ESI+ and m/z 554.2615 in ESI−) solution at 20 μl/min was used as the lock mass. Data were collected in centroid mode with a scan range of 50–1200 m/z, with lock mass scans collected every 30 seconds and averaged over 3 scans to perform mass correction. Additionally, data-dependent acquisition (DDA) of the QC sample was performed for structural elucidation for each ionisation mode.

Chromatograms and spectra were displayed using MassLynx (version 4.1; Waters Corp.). The MassLynx package ‘Databridge’ was used to convert the data files into NetCDF format. The package XCMS (R software) and an in-house developed script was used for the pre-processing of the data (peak-picking, alignment, grouping, zero filling, and median-fold change normalisation) [35]. Integrals of metabolic features with a coefficient of variation (CV) greater than 30% in the QC samples (*n* = 6) were removed in Microsoft Excel (2013, Microsoft, USA) prior to analysis.

### Statistical Analysis

Metabolic data from plasma ^1^H NMR spectra and lipid profiling UPLC-MS data were initially analysed in SIMCA software (V. 13.0, Umetrics) via Principal Component Analysis (PCA) [36], a non-supervised descriptive model used to investigate inherent similarities/differences across the data set and identify outliers. Analyses resulted in the removal of one NMR sample with an acquisition artefact thus bringing the final total to *n* = 66: 45 HAT and 21 controls (see S1 Table). PCA was additionally used to identify and remove contaminant/artefactual features, as described by Vorkas et al, 2015 [34], and to evaluate experimental reproducibility by assessing QC sample clustering for both ESI+ and ESI- runs. This was followed by Partial Least Squares Discriminant Analysis (PLS-DA), and Orthogonal PLS-DA (O-PLS-DA) [37]. Both are multivariate regression models used to assess the spectral differences that contribute towards classification of patients vs. controls, and to determine how well these differences can predict class separation. Contribution of within-class variation that is uncorrelated to the separation between classes is minimised in O-PLS-DA models, which were used for final extraction of discriminating metabolites. See supplementary protocols in supporting information (S1 File) for further details on data processing, analyses, and model validation.

For NMR data, metabolites integrals were then compared between HAT patients and controls via the two-tailed Welch’s (unequal variance) T-test in Microsoft Excel, followed by Benjamini-Hochberg multiple test correction [38]. Those where *p*<0.05 was observed were labelled as statistically significant discriminatory markers. For UPLS-MS data, threshold criteria for initial selection of markers were variables with a correlation value p(corr)[1]>0.5 and covariance p[1]>0.05, as visualized on the OPLS-DA S-plot, to extract the most discriminatory and robust features (see supporting protocols). This was followed by conducting a two-tailed Welch’s T-test with Benjamini-Hochberg correction, whereby the final statistical discriminatory criteria was also *p*<0.05 after multiple testing correction. Lipid species assignment of discriminatory features was based on the accurate mass-to-charge (m/z) ratio, searched against online MS database METLIN (https://metlin.scripps.edu) and previous published results generated in-house [34,39], as well as on the MS/MS information obtained in the DDA experiments of QC samples.

Integrals of these discriminatory variables were re-imported into SIMCA as new loadings for O-PLS-DA models, to evaluate the sensitivity and specificity of the combination of these metabolites for biomarker potential. O-PLS-DA models was performed twice, separately for NMR and UPLC-MS data. Firstly, models were generated using integrals of all significantly discriminatory markers, and secondly using only integrals of the top 5 discriminating markers, defined as those which displayed the greatest % difference between patients and controls. Calculations are further explained in supplementary protocols (see Supporting Information S1 File).

To explore links between potentially complementary metabolic datasets, NMR metabolite integrals were correlated with corresponding integral of discriminatory lipid profiling features from matching patient samples (*n* = 16), via Pearson correlation in MATLAB, followed by Benjamini-Hochberg correction. Similarly Pearson -based correlation analyses (with multiple test correction) was performed between metabolic (NMR and/or MS) data sets. Relationships between patient demographic data and metabolic datasets were assessed using Spearman correlation analysis, Mann-Whitney tests, and mixed linear models as appropriate, in JMP (v.10.0, SAS, USA).

## Results

### ^1^H NMR Spectroscopy Shows Marked Differences in Plasma between Human African Trypanosomiasis Patients and Controls

**Fig 1:**
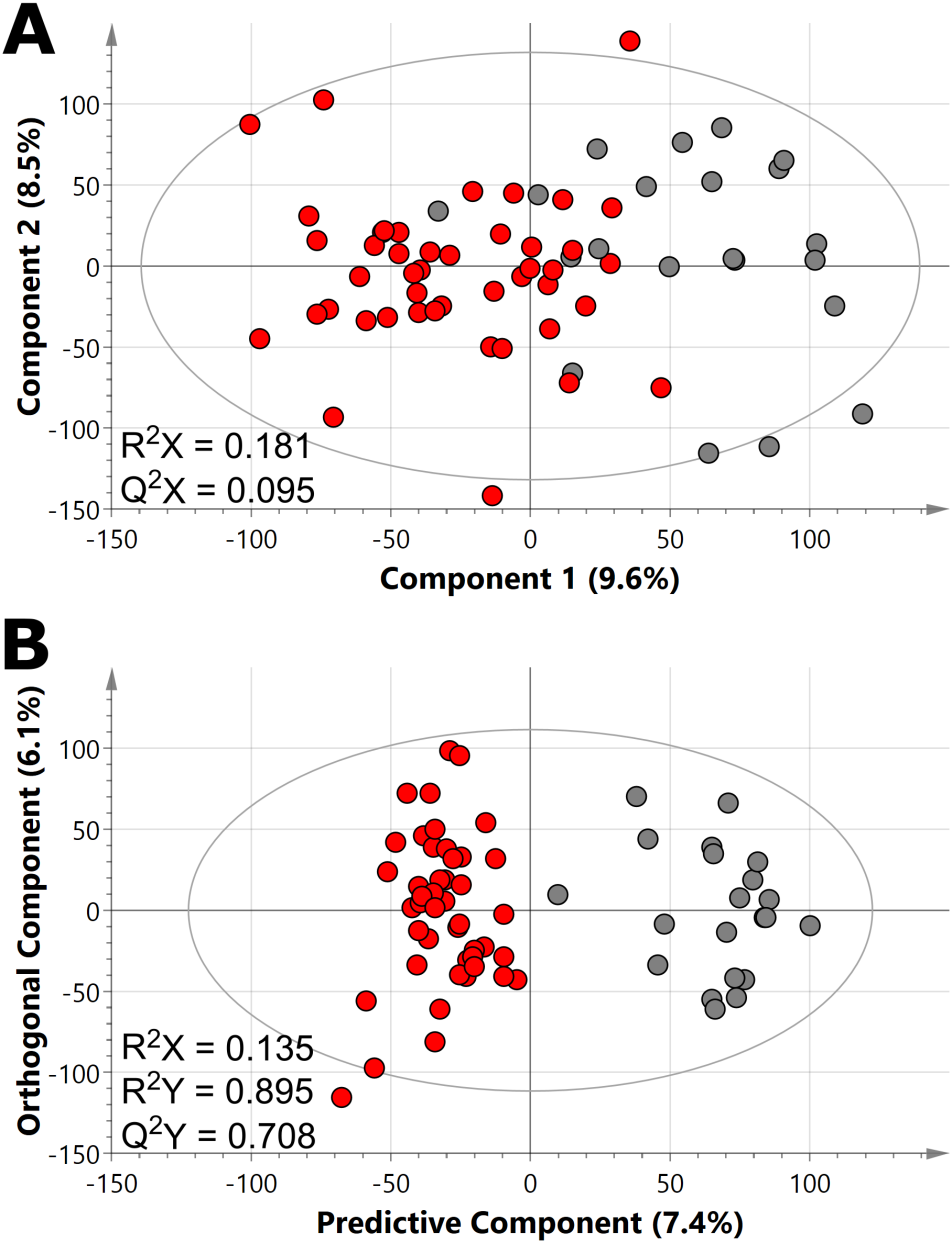
Plasma in HAT patients display different ^1^H NMR metabolic profiles compared with controls. PCA model (A) and O-PLS-DA model (B) score plots of plasma ^1^H NMR spectra across HAT patients and controls. Each circle represents a spectra from one sample, whereby patients are presented in red (*n* = 45) and controls in dark grey (*n* = 21). Abbreviations: R^2^X, model fit parameter for variation in spectral data; R^2^Y, model fit parameter for variation in classifier data (for O-PLS-DA); Q^2^, model predictive parameter for spectral data in PCA (Q^2^X) and for classifier data in O-PLS-DA (Q^2^Y). Individual component contribution of R^2^X are shown on the axes as percentage.

Inherent differences between *T*. *b*. *rhodesiense* infected HAT patients and uninfected controls dominated overall dataset variability, demonstrated by the partial separation of these two populations along the first component in PCA (Fig 1A), indicative that the HAT infection produced a strong metabolic response. No significant differences between metabolic profiles based on demographic factors (gender, age, or diagnostic stage) were found. Metabolic disparities between patients and controls were further examined using O-PLS-DA (a supervised modelling method that maximises differences between classes whilst minimising within-class variation), which showed complete segregation between patients and controls, also along the first component (Fig 1B). The model had a Q^2^Y (model predictive capability parameter) value of 0.708, indicating that the model was robust. Further validation through permutation testing found that the likelihood of achieving the same discriminatory result by chance was zero (*p* = 0 for both R^2^Y and Q^2^Y). It is noteworthy that although the first two components of the PCA model only cumulatively account for 18.1% of the total variance (R^2^) in the data set, indicating variability in sample composition, the O-PLS-DA model indicates that the metabolic signature is robust.

**Fig 2:**
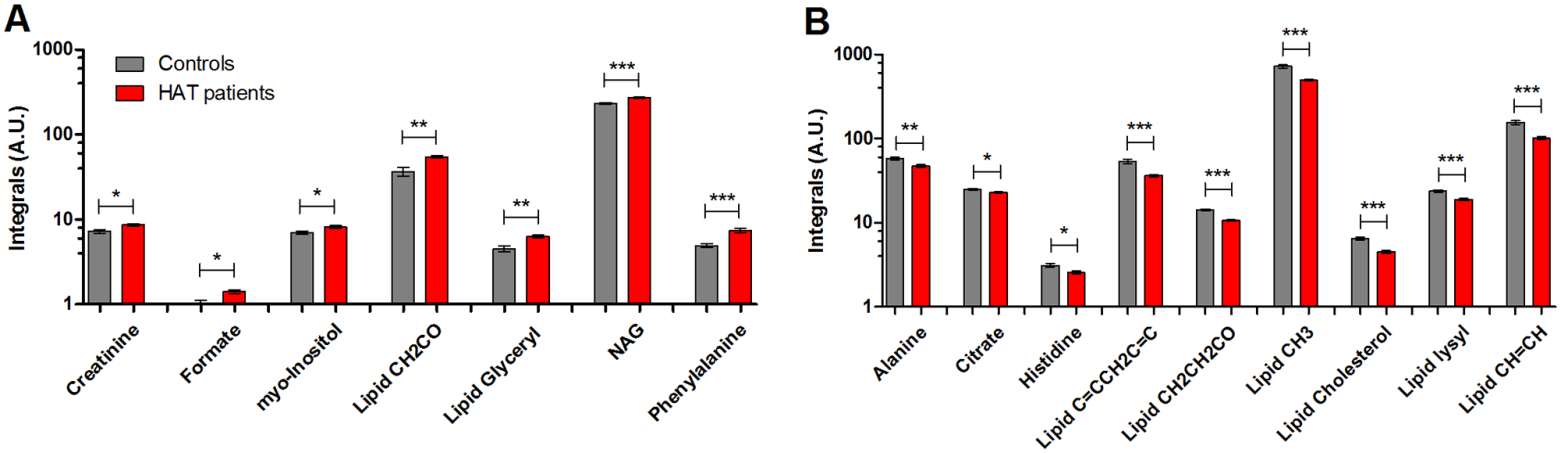
Differences in plasma metabolites between HAT patients and controls detected by NMR. Bar-charts show relative levels of plasma metabolites that were significantly altered between patients (shown in red, *n* = 45) and controls (shown in grey, *n* = 21), as measured by ^1^H NMR spectroscopy. (A) Metabolites higher in patients than controls. (B). Metabolites lower in patients than controls. Bars represent group mean average with standard error of the mean as error bars. Significance measured via Welch T-test with multiple test correction, shown as asterisks; * *p*<0.05, ** *p*<0.01, *** *p*<0.001. A.U., arbitrary units; NAG, *N*-acetyl glycoproteins.

Examination of the corresponding O-PLS-DA loadings revealed that the lipid peaks accounted for the majority of the discrimination. In total, 34 endogenous metabolites were identified from NMR spectra though only 31 of these displayed distinct peaks that were used for integral calculation (signals from isoleucine, leucine and lysine were superimposed by overlapping lipid peaks), and compared between patients and controls (shown in S2 Table). Those metabolites that were significantly higher in disease are shown in Fig 2A, whilst metabolites significantly lower in disease are shown in Fig 2B. The majority of lipid moieties detected in plasma were significantly reduced in patients, including those containing unsaturated bonds (CH = CH and C = CCH_2_C = C), lysyl group in plasma albumin, and cholesterol (Fig 2B). In contrast, lipid glyceryl and ketene (CH_2_CO) groups were significantly higher in patients than in controls (Fig 2A). Several plasma amino acids were also significantly discriminatory between HAT and controls, including histidine and alanine, which were found in lower concentrations in patients, and phenylalanine, which was augmented. Other significant changes included an increase of creatinine, *N*-acetyl glycoprotein, formate and *myo*-inositol in HAT patients whilst citrate was higher in controls. No associations between discriminatory metabolites and patient demographic data (age, gender or diagnostic stage) were found though the sample size in this study limited the power of this analysis (this was particularly the case for staging as our cohort had relatively few early cases—see S1 Table).

Additional O-PLS-DA models were built, firstly using integrals of all 16 discriminatory metabolites, followed by models using only the top 5 metabolites. The latter were defined as those which displayed the biggest measured percentage difference between HAT and controls, representing metabolites of most practical diagnostic potential. These consisted of phenylalanine, formate and lipid moieties CH_2_CO, CH = CH, and glyceryl. In both O-PLS-DA models, results provided identical specificity scores for classifying patients and controls samples, namely 95.24% (with 95% confidence intervals [CI] of 86.1–100%). Model sensitivity was 88.89% (79.7–98.1% CI) using all NMR discriminatory markers, and 84.44% (73.9–95.0% CI) using just the top 5 metabolites (see S3 Table for confusion matrices).

### Lipid Profiling UPLC-MS Reveals Compositional Differences in Plasma Lipid Species between HAT and Controls

Lipid plasma profiles were further examined using reversed-phase UPLC-MS. In total, 8771 metabolic features were detected in positive ionisation mode (ESI+), whilst 2543 features were observed in negative mode (ESI-). Following the exclusion of peaks with CV > 30% across the QC’s, as well as the removal of contaminant peaks present in either QC’s or blank controls, 6952 features in ESI+ and 1714 features in ESI- were retained for final analyses. PCA models showed tight clustering of QC samples compared with the remaining test samples (supplementary information S1 Fig), assuring the robustness of the MS experimental runs.

**Fig 3:**
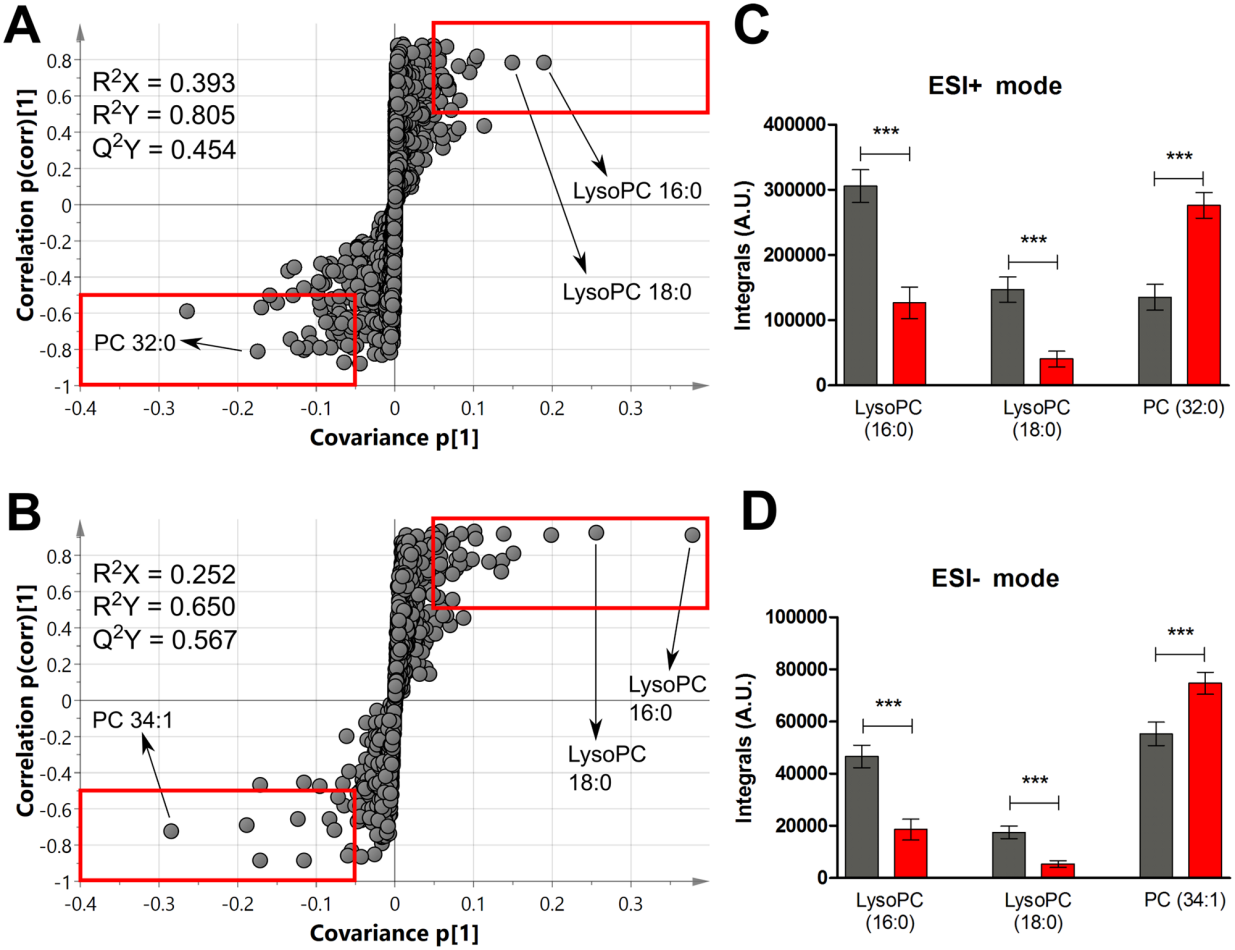
Plasma in HAT patients display different lipid profiles compared with controls. S-plots of O-PLS-DA model for plasma lipid profiling UPLC-MS features detected in ESI+ mode (A) and ESI- mode (B), whereby each circle represents one feature with a unique combination of m/z and retention time values. Discriminatory features selected surpassed p[1] and p(corr)[1] threshold criteria, highlighted in red boxes (see Methods). See S1 Fig for corresponding scores plots. Integrals of features highlighted in the S-plots are shown as bar-chart showing mean averages ± standard error of the mean, for both ESI+ (C) and ESI- (D). Patients levels are shown in red (*n* = 16) and controls in dark grey (*n* = 14). Significant differences, as measured via Welch T-test with multiple test correction, are labelled with asterisks, where *** *p*<0.001. Abbreviations: A.U., arbitrary units; LysoPC, lysophosphatidylcholine; PC, phosphatidylcholine.

As noted with NMR, lipid profiling results showed an inherent separation between the two classes already in the first two components of PCA, observed in both ionisation modes (see S1 Fig). These differences were confirmed in the corresponding O-PLS-DA models (S1 Fig). Permutation testing confirmed model robustness (ESI+, *p* = 0.004 for R^2^Y and *p* = 0 for Q^2^Y and for ESI-, *p* = 0 for both R^2^Y and Q^2^Y). Metabolic features responsible for class discrimination (i.e. HAT patients vs. controls) were visualised in S-plots in SIMCA, with the cut-off values of p(corr)[1]>0.5 and covariance p[1]>0.05 were used as selection criteria (see Methods), shown in Fig 3A and 3B. Integrals of selected discriminatory features highlighted in the S-plots are shown in Fig 3C and 3D, demonstrating significant decreases in lysophosphatidylcholines 16:0 and 18:0, as well as increases in phosphatidylcholine 32:0 and 34:1.

Overall, lipid profiling analysis identified 37 unique features that were statistically different between HAT and controls, 12 of which were found to be significantly different in both of these modes (Table 1). Features included six lysophosphatidylcholines (LysoPCs 16:0, 18:0, 18:1, 18:2, 20:3 and 20:4), four phosphatidylcholines (PCs 32:0, O-34:1, 38:3 and 38:5) and two sphingomyelin species (SMs d40:1 and d41:1). Further information on UPLC-MS assignments of features can be found in S4 Table. The majority of lipid classes studied were glycerophospholipids (LysoPCs and PCs), followed by sphingolipids (mainly sphingomyelins) and triglycerides (see Table 1 and S4 Table). Free fatty acids were detected using our MS methodology but did not significantly differ between patients and controls.

**Table 1:** Discriminatory lipid species observed in plasma via UPLC-MS.

	Lipid Species	ESI+		ESI-	
		P-value	% Difference	P-value	% Difference
**Increased in HAT**	Cer (d42:2)	-	-	2.51E-03	54.30%
	PC (32:0)	2.49E-05	104.20%	2.58E-03	111.70%
	PC (32:1)	-	-	1.29E-04	62.50%
	PC (34:1)	-	-	8.21E-03	35.00%
	PC (34:2)	-	-	1.29E-04	40.20%
	PC(O-34:1)/ PC(P-34:0)	3.93E-04	86.20%	2.00E-03	84.30%
	PE (36:2)	-	-	2.51E-03	54.40%
	PS (39:3)	-	-	4.42E-03	37.50%
	SM (d34:1)	1.75E-02	96.10%	-	-
	TG (51:2)	6.52E-05	98.00%	-	-
	TG (52:3)	4.40E-03	45.40%	-	-
	TG (53:2)	6.73E-04	86.10%	-	-
	TG (53:3)	4.02E-04	77.50%	-	-
	TG (54:2)	3.74E-04	70.30%	-	-
	TG (54:5)	2.20E-03	68.20%	-	-
**Decreased in HAT**	CE (18:2)	1.12E-04	-53.10%	-	-
	LysoPC (16:0)	1.93E-05	-58.60%	5.42E-04	-60.10%
	LysoPC (18:0)	1.11E-04	-72.60%	4.42E-03	-69.50%
	LysoPC (18:1)	1.98E-05	-66.50%	1.00E-04	-62.50%
	LysoPC (18:2)	3.28E-04	-80.50%	3.18E-04	-78.20%
	LysoPC (20:3)	5.97E-05	-75.00%	7.00E-04	-77.90%
	LysoPC (20:4)	4.36E-06	-75.60%	2.62E-04	-73.10%
	PC (40:5)	1.13E-02	-39.20%	-	-
	PC (36:3)	6.56E-03	-33.90%	-	-
	PC (38:3)	1.93E-03	-43.00%	2.51E-03	-30.70%
	PC (38:5)	1.71E-02	-40.90%	1.29E-04	-28.80%
	PC (38:6)	1.63E-02	-33.30%	-	-
	PC (40:4)	3.25E-03	-47.90%	-	-
	PC (40:6)	2.94E-02	-32.70%	-	-
	PC(O-34:3)/PC(P-34:2)	8.43E-04	-52.80%	-	-
	SM (d38:1)	-	-	3.46E-03	-37.60%
	SM (d39:1)	-	-	4.42E-03	-52.10%
	SM (d40:1)	1.01E-03	-43.90%	3.46E-03	-48.60%
	SM (d40:2)	-	-	2.58E-03	-60.20%
	SM (d41:1)	1.06E-03	-58.60%	2.00E-03	-48.20%
	SM (d41:2)	-	-	4.42E-03	-55.30%
	SM (d42:1)	-	-	3.92E-04	-52.50%

Molecular species that not detected in a particular electro-spray ionisation (ESI) mode were omitted from the table. For more description of detected lipid species (including retention times and mass/charge ratios) see supplementary S2 Table.

Abbreviations: -, no discriminatory marker detected that ionisation mode; Cer, ceramide; CE, cholesterol ester; LysoPC, lysophosphatidylcholine; LysoPE, lysophosphatidylethanolamine; NS, not statistically significant; PC, phosphatidylcholine; PE, phosphatidylehtanolamine; PS, phosphatidylserine; RT, retention time; SM, sphingomyelin.

In total, six significantly altered lysoPCs measured in this study were found to be lower in patients than controls. Significantly lower intensities were also observed for eight out of nine sphingolipids, including seven sphingomyelins (SM) as well as nervonic ceramide (Cer d42:2). Only SM d34:1, the smallest of the analysed SM species, was found to be higher in patients than controls. Additionally, patients had augmented levels of six triglycerides (TG) species and depleted levels of cholesterol ester (CE) 18:2 in patients, in line with NMR findings where we detected higher glyceryl moieties and lower cholesterol in HAT.

In general, differences in lipid species concentrations between patients and controls were similar across the lipid subclasses, with the exception of the phosphatidylcholines. In contrast, our UPLC-MS results indicate that PCs that contained a high number of saturated bonds were significantly decreased in patients (e.g. PCs 38:5, 38:6, 40:5), whilst those with two or fewer unsaturated bonds were increased (e.g. PCs 32:0, 32:1, and 34:1, see Table 1). These results mirror the NMR findings, where lipid moieties with unsaturated bonds (CH = CH and C = CCH2C = C) were significantly lower in patients than controls (Fig 2).

Similar to the NMR data, additional O-PLS-DA models were generated using the integrals all 37 markers from both ionisation modes, followed by further models using only the top 5 features with the greatest % difference (as shown in Table 1). As most of the largest changes were observed in the positive mode, we selected the feature from this ionisation mode only. These included PC(34:3), PC(32:0), TG(51:2), PC(O-34:1) and SM(d34:1). Both O-PLS-DA models with either all markers or top 5 gave identifical sensitivity and specificity scores, namely 93.75% (81.9–100% CI) and 85.71% respectively (67.4–100% CI; see S5 Table for confusion matrices).

Potential links between UPLC-MS and NMR data was explored by correlating lipid metabolic features with NMR metabolite levels measured via NMR. Whilst multiple trends were observed, including links between lipid MS species and several NMR lipid moieties, 3-hydroxybutyrate (a ketone body generated from fatty acid break-down), and energy associated metabolites, e.g. creatinine and lactate), none of these associations remained statistically significant after Benjamini-Hochberg multiple test correction was performed, due to lack of statistical power. No significant links were observed between MS data and age and/or gender data (analyses could not be performed for diagnostic staging due to limited numbers of early stage within this subset of data—see S2 Table).

## Discussion

The HAT infection was characterised by strong metabolic differences in plasma profiles, based on both NMR and UPLC-MS, which shows the potential of this approach in the development of novel diagnostic marker sets. The discriminatory profile was largely driven by variation in lipids and amino acids. Phenylalanine was the single most discriminatory metabolite as detected by NMR, with the largest percentage difference between HAT and controls in concentration. Raised levels of phenylalanine have been frequently linked with infection and inflammation [40]. Phenylalaninemia has previously been reported in patients with enteric fever [41], and with malaria [42] Evidence suggests that this phenomenon may be related to neurological effects seen in cerebral malaria [42], since high levels of phenylalanine are known to be neurotoxic [43]. Other differences in plasma amino acid concentrations included significant decreases in histidine and alanine in HAT. Histidine concentrations have been shown to be negatively correlated with inflammatory disease markers in humans [44–46] and this would be consistent with clinical evidence of systemic inflammatory responses in HAT [3].

Creatinine concentration was also found to be significantly higher in HAT, potentially a sign of renal dysfunction which is one of the clinical features observed in sleeping sickness [47,48]. Additionally, concentrations of *N*-acetyl groups of plasma glycoproteins were also higher in patients. Most of these have been shown to belong to α1 acid glycoprotein [49,50], another inflammatory marker known to be associated with the acute-phase response. This result is similar to what has been found with experimental murine infection of *T*. *b*. *brucei* where plasma acetylated glycoproteins (dominated by *O*-acetyls) were found to be augmented [11].

Some of the most pronounced changes observed in the NMR spectra were the clear disparities in the plasma lipid composition between the two groups. In addition to their roles as energy reserves and major constituents of membranes, lipids also represent highly biologically active metabolites, with involvement (or function) in signalling and a range of inflammatory processes, e.g. prostaglandins and leukotrienes synthesis [51]. Our results showed that the majority of the concentrations of discriminatory lipids were significantly reduced in patients. These primarily included lipids containing unsaturated bonds (CH = CH and C = CCH_2_C = C), CH_3_ and CH_2_CH_2_CO moieties, and albumin lysyl groups, and high-density lipoprotein (HDL) cholesterol. Dyslipidaemia has been observed in a range of infections, measured primarily using lipid-specific biochemical serum assays, as opposed to comprehensive metabolic profiling, whereby significantly reduced HDL and total cholesterol have been reported in malaria [52], enteric fever [53] and others [54,55]. Decreased lipoprotein CH_3_ moieties were also observed in experimental murine *T*. *b*. *brucei* infection [11], confirming the need for deeper assessment into the variety of lipid species, to help dissect their different roles during infection.

Further investigation of the lipid profile with UPLC-MS indicated that HAT patients displayed significantly lower levels of polyunsaturated phosphatidylcholines (e.g. PC 40:6, 40:5, 38:5 and 38:3), although PC molecules with 2 or fewer unsaturated bonds were in contrast increased. This profile presents a more complex picture than was obtained in experimental mouse infections with *T*. *b*. *brucei*, where an overall decrease in plasma PC concentrations was measured by NMR [11].

A range of lysophophatidylcholines (e.g. LysoPC 16:0, 18:0, 18:2) intensities was also significantly lower in patients. Despite the fact that *Trypanosoma brucei ssp* have the capability to produce many of their lipids *de novo*, they are known to scavenge several lipid precursor molecules from the host and have been shown to require lipid uptake for optimal growth [56,57]. More specifically, these include fatty acids and lyso-phospholipids, which are assembled to make phospholipids, the most dominant lipid component of *T*. *brucei ssp* [58]. As fatty acyl chain composition in *T*. *b*. *rhodesiense* bloodstream forms have a high proportion of long-chain polyunsaturated fatty acids [59,60], it could be hypothesised that the lower plasma concentrations of polyunsaturated lipids in HAT patients may be due to increased uptake by the parasites.

Numerous sphingomyelin species were also found in lower concentrations in infection (e.g. SM d40:1, d41:1 and d42:1), accompanied by a significant increase of ceramide. The sphingolipid turnover is regulated by the balance between enzymatic degradation and synthesis, with ceramide being a major break-down product. Oxidative stress, inflammatory cytokines and infection can trigger sphingomyelinase enzymes to increase ceramide generation. Higher levels of ceramide, in turn, have been linked to increased cell autophagy and apoptosis (the latter particularly in endothelial cells leading to increased vascular permeability), additional pro-inflammatory cytokine and chemokine synthesis, and other metabolic disorders (e.g. insulin resistance and obesity) [61]. Thus, the changes in sphingolipids observed in HAT further highlights the widespread inflammatory effects on the host, associated with *T*. *b*. *rhodesiense* infection.

Another characteristic of HAT patients was hypertriglyceridaemia. Not only is this result is in agreement with a range of rabbit and non-human primate models of *T*. *brucei spp* infections [21,22], but is also consistent with marked increases in serum triglycerides in *T*. *b*. *gambiense* HAT [62]. However, the disease discriminatory potential of hypertriglyceridaemia (often combined with increases in circulating free fatty acids) will require further investigation as it has been shown to be a common feature across numerous infections in humans [52–54].

In summary, the presented work is the first to apply a comprehensive metabolic profiling approach to the investigation of *Trypanosoma brucei* infection in humans and highlights the potential of metabonomic technology for developing a diagnostic platform for HAT. The models generated based on both NMR and UPLC-MS were robust, thus the utility of this technology in an exploratory capacity is evident. Similarities but importantly significant differences in discriminatory NMR metabolites were identified to previously published metabolic profiles in experimental rodent *T*. *brucei spp*. infections. We identified a range of discriminatory plasma metabolites in clinical HAT that collectively have diagnostic disease marker potential (up to 93.75% sensitivity and 95.24% specificity). While some of these markers have been associated with other infections or inflammatory processes, the effects of specific infection on a combined panel such as in this study has not previously been reported and may prove diagnostically effective in differentiating HAT from other endemics disease. However, it is obvious that neither NMR spectroscopy nor UPLC-MS are platforms that can be used at point-of-care in developing countries. Therefore the functionality of these platforms lies in characterisation of the metabolic consequences of a parasitic infection, with subsequent development of simpler clinical assays such as a biochemical dipstick based on the strongest differentiating biomarkers. Of particular interest from our results were differences in lipids, observed in both NMR and UPLC-MS, that dominated the variation in metabolic profiles between HAT and controls. Further investigation of inflammatory lipid mediators such prostaglandins and leukotrienes would also be of particular interest, to better understand pathology and provide key insights into links between lipid metabolism and immune responses in the host. Detailed lipid sub-species characterisation with the use of lipid reference standards and MS/MS are required to further isolate novel diagnostic candidates to ultimately aid in the detection and management of *T*. *b*. *rhodiesiense* HAT, moving closer towards the WHO goal of disease eradication.

## Supporting Information

S1 Fig Differences observed in plasma lipid-MS profiles between HAT patients and controls. Multivariate model scores plots based on lipid MS features of plasma samples. PCA models based on first two component of all plasma test samples (as light grey circles, *n* = 30) and QC’s (as blue circles, *n* = 6) in positive ionisation (ESI+) mode (A) and negative (ESI-) mode (B). PCA models of the first two components of plasma test samples only, comparing HAT patients (red circles, *n* = 16) vs. controls (dark grey circles, *n* = 14), in ESI+ (C) and ESI- (D). O-PLS-DA models of test samples comparing HAT vs. controls (labelling and group sizes as described for PCA), in ESI+ (E) and ESI- (F). O-PLS-DA based on both predictive and orthogonal components for ESI+ but only on the first predictive component for ESI- (the orthogonal component for this model reduced the predictive ability of model and was thus omitted). Abbreviations: R^2^X, model fit parameter for variation in spectral data; R^2^Y, model fit parameter for variation in classifier data (for O-PLS-DA models); Q^2^, model predictive parameter (for spectral data Q^2^X in PCA/for classifier data Q^2^Y in O-PLS-DA), SD, standard deviation.
(TIF)

S1 Table Patient and control cohort demographic information. (PDF)

S2 Table Plasma metabolite levels measured via ^1^H NMR spectroscopy. (PDF)

S3 Table Confusion matrices of HAT vs. control classification ability of NMR discriminatory markers. (PDF)

S4 Table List of Discriminatory Lipid Species detected via UPLC-MS in both ionisation modes. (PDF)

S5 Table Confusion matrices of HAT vs. control classification ability of NMR discriminatory markers. (PDF)

S1 File Supplementary protocols for statistical analyses. (DOCX)
