# Supplementary figures and images for: Discovery of Infection Associated Metabolic Markers in Human African Trypanosomiasis
Source: PLoS Negl Trop Dis. 2015 Oct 27;9(10):e0004200. doi: 10.1371/journal.pntd.0004200 (PMC4624234; doi:10.1371/journal.pntd.0004200)

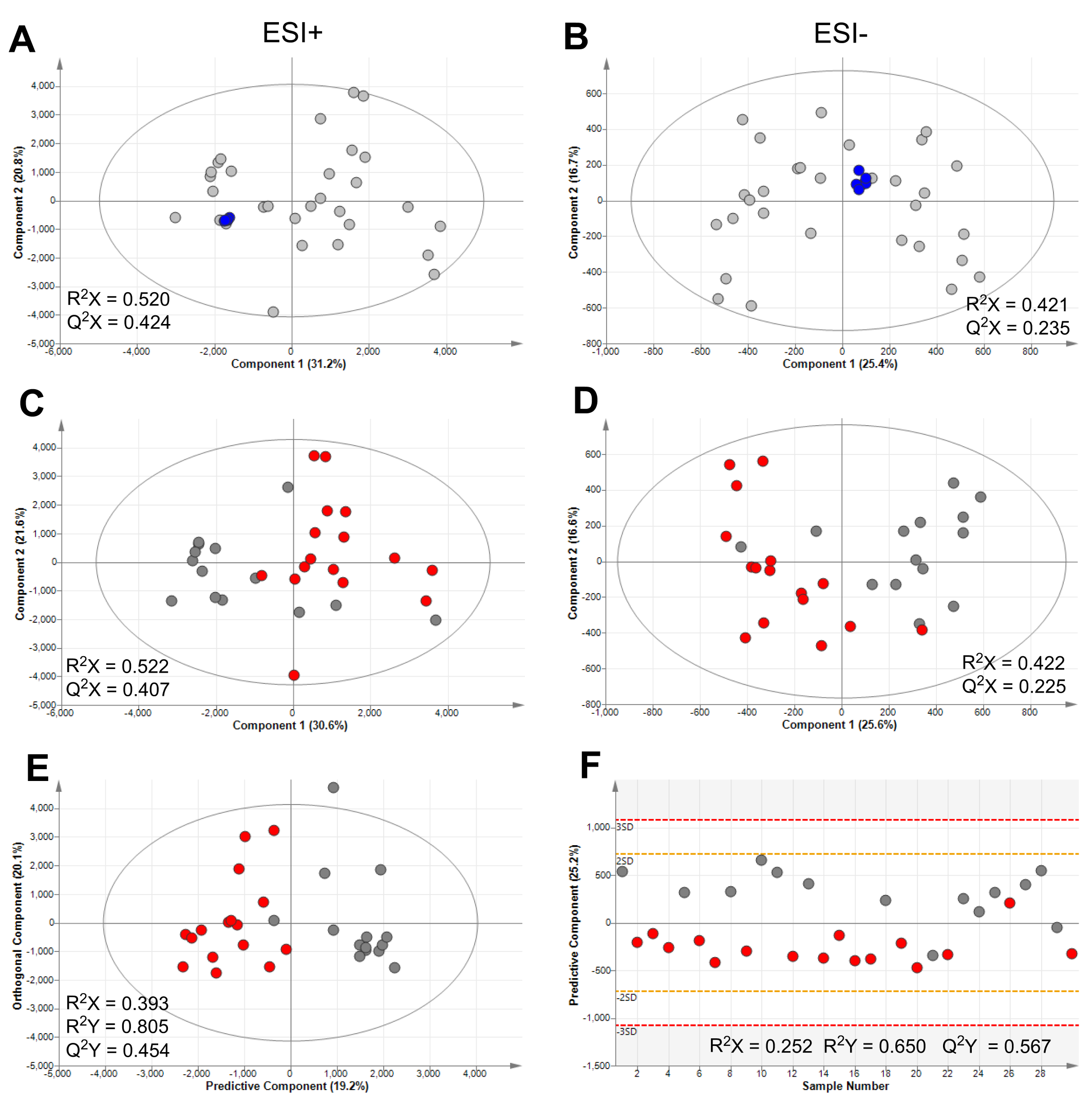

Supplement: S1 Fig — Multivariate model scores plots based on lipid MS features of plasma samples. PCA models based on first two component of all plasma test samples (as light grey circles, n = 30) and QC’s (as blue circles, n = 6) in positive ionisation (ESI+) mode (A) and negative (ESI-) mode (B). PCA models of the first two components of plasma test samples only, comparing HAT patients (red circles, n = 16) vs. controls (dark grey circles, n = 14), in ESI+ (C) and ESI- (D). O-PLS-DA models of test samples comparing HAT vs. controls (labelling and group sizes as described for PCA), in ESI+ (E) and ESI- (F). O-PLS-DA based on both predictive and orthogonal components for ESI+ but only on the first predictive component for ESI- (the orthogonal component for this model reduced the predictive ability of model and was thus omitted). Abbreviations: R2X, model fit parameter for variation in spectral data; R2Y, model fit parameter for variation in classifier data (for O-PLS-DA models); Q2, model predictive parameter (for spectral data Q2X in PCA/for classifier data Q2Y in O-PLS-DA), SD, standard deviation. (TIF) [file pntd.0004200.s001.tif]
