# Supplementary material for: Discovery of Infection Associated Metabolic Markers in Human African Trypanosomiasis
Source: PLoS Negl Trop Dis. 2015 Oct 27;9(10):e0004200. doi: 10.1371/journal.pntd.0004200 (PMC4624234; doi:10.1371/journal.pntd.0004200)
Supplement: S1 Table — (PDF) [file pntd.0004200.s002.pdf]

S1 Table. Patient and control cohort demographic information

	**Total Samples (*n*=67)**		**Final Samples in NMR Data (*n*= 66)**		**Final Subset of Samples in UPLC-MS Data (*n*=30)**	
	**Controls (*n*=21)**	**HAT Patients (*n*=46)**	**Controls (*n*=21)**	**HAT Patients (*n*=45)**	**Controls (*n*=14)**	**HAT Patients (*n*=16)**
Age, median (IQR) in years	31 (22-50)	26 (17-43)	31 (22-50)	26 (17-44)	32 (24-54)	41 (22-55)
Sex, number of males: females	10:11	19:27	10:11	19:26	7:7	7:9
Diagnostic Stage, number of early/total	N/A	10/46	N/A	10/45	N/A	3/16

Abbreviations: IQR, Inter-quartile range; N/A, not applicable; There were no significant differences in age distribution or gender ratio between patient and control groups or the subsets that were analysed.
