# Supplementary material for: Discovery of Infection Associated Metabolic Markers in Human African Trypanosomiasis
Source: PLoS Negl Trop Dis. 2015 Oct 27;9(10):e0004200. doi: 10.1371/journal.pntd.0004200 (PMC4624234; doi:10.1371/journal.pntd.0004200)
Supplement: S2 Table — (PDF) [file pntd.0004200.s003.pdf]

S2 Table. Plasma metabolite levels measured via ^{1}H NMR spectroscopy

Metabolite	Integrated Peak (multiplicity)	Average Integral in Controls (± SEM) in A.U.	Average Integral in HAT (± SEM) in A.U.	% Difference (HAT - Control)	P-value
3-Hydroxybutyrate	2.32(m)	29.66 (± 0.89)	32.09 (± 1.31)	8.2%	NS
Acetate	1.94(s)	6.60 (± 0.38)	6.71 (± 0.46)	1.7%	NS
Acetoacetate	2.25(s)	8.93 (± 0.72)	10.85 (± 0.85)	21.5%	NS
Alanine	1.49(d)	57.65 (± 2.18)	46.84 (± 1.97)	-18.7%	1.60E-03
Citrate	2.68(d)	24.97 (± 0.64)	22.87 (± 0.43)	-8.4%	2.01E-02
Creatine	3.94(s)	8.17 (± 0.38)	10.24 (± 0.85)	25.4%	NS
Creatinine	4.07(s)	7.26 (± 0.35)	8.61 (± 0.30)	18.7%	1.30E-02
Formate	8.47(s)	1.00 (± 0.13)	1.40 (± 0.08)	41.0%	2.13E-02
Glucose	5.25(s)	76.87 (± 4.92)	79.49 (± 3.71)	3.4%	NS
Glutamine	2.14(m)	67.08 (± 2.09)	67.64 (± 1.58)	0.8%	NS
Glycerophosphocholine	4.32(m)	19.97 (± 1.06)	18.27 (± 0.33)	-8.5%	NS
Glycine	3.58(s)	21.79 (± 1.34)	19.20 (± 0.68)	-11.9%	NS
Histidine	7.08(s)	3.12 (± 0.16)	2.58 (± 0.10)	-17.4%	1.79E-02
Lactate	1.34(d)	531.15 (± 97.44)	503.81 (± 55.89)	-5.1%	NS
Lipid $C=CCH_2C=C$	2.78(m)	53.59 (± 2.71)	36.07 (± 1.08)	-32.7%	1.35E-05
Lipid CH_3	0.88(m)	718.89 (± 33.00)	495.75 (± 13.50)	-31.0%	8.46E-06
Lipid CH_2-C=C	2.02(m)	161.76 (± 7.13)	147.48 (± 3.07)	-8.8%	NS
Lipid CH_2CH_2CO	1.51(m)	14.21 (± 0.39)	10.63 (± 0.20)	-25.2%	5.28E-08
Lipid CH_2CH_2-C=C	1.58(m)	59.26 (± 7.19)	74.80 (± 2.74)	26.2%	NS
Lipid CH_2CO	2.23(m)	36.29 (± 4.35)	54.84 (± 1.89)	51.1%	1.60E-03
Lipid CH_2	1.28(m)	992.66 (± 79.79)	981.73 (± 33.16)	-1.1%	NS
Lipid Cholesterol (HDL)	0.68(m)	6.50 (± 0.21)	4.52 (± 0.15)	-30.4%	5.28E-08
Lipid Glyceryl CH	5.20(m)	4.53 (± 0.39)	6.28 (± 0.22)	38.8%	1.48E-03
Lipid ε-CH_2 lysyl (albumin)	2.93(m)	23.85 (± 0.81)	18.82 (± 0.49)	-21.1%	2.53E-05
Lipid, unsaturated CH=CH	5.30(m)	154.75 (± 8.53)	101.50 (± 3.89)	-34.4%	2.10E-05
myo-Inositol	4.087(t)	6.98 (± 0.32)	8.21 (± 0.29)	17.6%	1.58E-02
N-acetyls of glycoproteins	2.06(m) & 2.09(m)	233.03 (± 4.68)	270.13 (± 4.67)	15.9%	7.02E-06
Phenylalanine	7.45(t)	4.92 (± 0.25)	7.44 (± 0.46)	51.2%	4.18E-05
Pyruvate	2.38(s)	17.64 (± 2.05)	26.99 (± 4.27)	53.0%	NS
Tyrosine	6.92(d)	6.19 (± 0.38)	6.87 (± 0.31)	11.0%	NS
Valine	1.05(d)	21.90 (± 1.37)	22.10 (± 0.79)	0.9%	NS

Table lists metabolites that were identified in plasma ^{1}H NMR spectra and spectral regions used for integral calculation and Welch T-test analyses. * % Difference calculated relative to control group average. Peaks from isoleucine, leucine and lysine were omitted as predominant peaks were superimposed by larger lipids signals. Abbreviations: A.U., arbitrary units; (d), doublet; (m), multiplet; NS, not significant; (s), singlet; SEM, standard error of the mean; (t), triplet.
