# Supplementary material for: Discovery of Infection Associated Metabolic Markers in Human African Trypanosomiasis
Source: PLoS Negl Trop Dis. 2015 Oct 27;9(10):e0004200. doi: 10.1371/journal.pntd.0004200 (PMC4624234; doi:10.1371/journal.pntd.0004200)
Supplement: S3 Table — (PDF) [file pntd.0004200.s004.pdf]

S3 Table. Confusion matrices of HAT vs. control classification ability of NMR discriminatory markers

	**Using all 16 Discriminatory Markers**		**Using Top 5 Discriminatory Markers**	
	**Actual HAT Patients**	**Actual Controls**	**Actual HAT Patients**	**Actual Controls**
Predicted HAT Patients	40 (TP)	1 (FP)	38 (TP)	1 (FP)
Predicted Controls	5 (FN)	20 (TN)	7 (FN)	20 (TN)
	Sensitivity = TP/[TP+FN] = 88.89%	Specificity = TN/[TN+FP] = 95.24%	Sensitivity = TP/[TP+FN] = 84.44%	Specificity = TN/[TN+FP] = 95.24%
Upper 95% CI	98.07%	100%*	95.03%	100%*
Lower 95% CI	79.71%	86.13%	73.86%	86.13%

Classification based on O-PLS-DA models, as described in supplementary protocols in Supporting Information (for 16 Markers: R^2Y 0.736, Q^2Y 0.657; for top 5 markers: R^2Y 0.641, Q^2Y 0.582). Abbreviations: *calculated upper CI was capped to absolute maximal value of 100%; CI, confidence interval; FN, false negatives; FP, false positives; TN, true negatives; TP, true positives.
