# Supplementary material for: Discovery of Infection Associated Metabolic Markers in Human African Trypanosomiasis
Source: PLoS Negl Trop Dis. 2015 Oct 27;9(10):e0004200. doi: 10.1371/journal.pntd.0004200 (PMC4624234; doi:10.1371/journal.pntd.0004200)
Supplement: S4 Table — (PDF) [file pntd.0004200.s005.pdf]

S4 Table. List of Discriminatory Lipid Species detected via UPLC-MS in both ionisation modes

Lipid Class	Lipid Subclass	Composition (total Carbons: unsaturated bonds)	Chemical Formula	Observed Ion	RT (mins)	Predicted neutral mass	Measured m/z	Mass Accuracy (ppm)	P-value	% Difference (HAT – Control)
ESI Positive Mode										
Sterol Lipids	CE	18:2	C45H76O2	[M+NH4]+	15.70	648.585	666.621	3	1.12E-04	-53.1%
Glycero-phospholipids	LysoPC	16:0	C24H50NO7P	[M+H]+	1.23	495.333	496.340	1	1.93E-05	-58.6%
Glycero-phospholipids	LysoPC	18:1	C26H52NO7P	[M+H]+	1.29	521.348	522.355	0	1.98E-05	-66.5%
Glycero-phospholipids	LysoPC	18:0	C26H54NO7P	[M+H]+	1.70	523.364	524.372	1	1.11E-04	-72.6%
Glycero-phospholipids	LysoPC	18:2	C26H50NO7P	[M+H]+	1.05	519.333	520.338	3	3.28E-04	-80.5%
Glycero-phospholipids	LysoPC	20:3	C28H52NO7P	[M+H]+	1.55	545.348	546.355	0	5.97E-05	-75.0%
Glycero-phospholipids	LysoPC	20:4	C28H50NO7P	[M+H]+	1.03	543.333	544.340	0	4.36E-06	-75.6%
Glycero-phospholipids	PC	32:0	C40H80NO8P	[M+H]+	7.69	733.562	734.572	3	2.49E-05	104.2%
Glycero-phospholipids	PC	32:0	C40H80NO8P	[M+Na]+	7.70	733.562	756.555	1	3.68E-06	135.2%
Glycero-phospholipids	PC	O-34:1/ P-34:0	C42H84NO7P	[M+H]+	9.32	745.599	746.607	1	3.93E-04	86.2%
Glycero-phospholipids	PC	O-34:3 /P-34:2	C42H80NO7P	[M+H]+	7.24	741.567	742.576	1	8.43E-04	-52.8%
Glycero-phospholipids	PC	36:3	C44H82NO8P	[M+H]+ isotope	6.89	783.578	785.591	1	6.56E-03	-33.9%
Glycero-phospholipids	PC	38:3	C46H86NO8P	[M+H]+ isotope	9.16	811.609	813.622	5	1.93E-03	-43.0%
Glycero-phospholipids	PC	38:5	C46H82NO8P	[M+H]+	6.24	807.578	808.587	2	1.71E-02	-40.9%
Glycero-phospholipids	PC	38:6	C46H80NO8P	[M+H]+	6.15	805.562	806.573	4	1.63E-02	-33.3%
Glycero-	PC	40:4	C48H88NO8P	[M+H]+	9.95	837.625	838.635	3	3.25E-03	-47.9%

phospholipids										
Glycero-phospholipids	PC	40:5	C48H86NO8P	[M+H]+	8.22	835.609	836.619	3	1.13E-02	-39.2%
Glycero-phospholipids	PC	40:6	C48H84NO8P	[M+H]+	7.53	833.594	834.603	2	2.94E-02	-32.7%
Sphingolipids	SM	d34:1	C39H79N2O6P	[M+H]+	5.52	702.568	703.576	1	1.75E-02	96.1%
Sphingolipids	SM	40:1	C45H91N2O6P	[M+H]+	12.90	786.662	787.672	4	1.01E-03	-43.9%
Sphingolipids	SM	d41:1	C46H93N2O6P	[M+H]+	13.22	800.677	801.687	3	1.06E-03	-58.6%
Glycerolipids	TG	51:2	C54H100O6	[M+NH4]+	15.73	844.752	862.791	6	6.52E-05	98.0%
Glycerolipids	TG	52:3	C55H100O6	[M+NH4]+	15.60	856.752	874.791	5	4.40E-03	45.4%
Glycerolipids	TG	53:2	C56H104O6	[M+NH4]+	15.99	872.783	890.821	4	6.73E-04	86.1%
Glycerolipids	TG	53:3	C56H102O6	[M+NH4]+	15.74	870.768	888.806	5	4.02E-04	77.5%
Glycerolipids	TG	54:2	C57H106O6	[M+NH4]+	16.13	886.799	904.835	2	3.74E-04	70.3%
Glycerolipids	TG	54:5	C57H100O6	[M+NH4]+	15.51	880.752	898.789	3	2.20E-03	68.2%
ESI Negative Mode										
Sphingolipids	Cer	d42:2	C42H81NO3	[M+FA-H]-	13.62	647.622	692.618	2	2.51E-03	54.3%
Glycero-phospholipids	LysoPC	16:0	C24H50NO7P	[M+FA-H]-	1.23	495.333	540.329	1	5.42E-04	-60.1%
Glycero-phospholipids	LysoPC	18:0	C26H54NO7P	[M+FA-H]-	1.70	523.364	568.360	3	4.42E-03	-69.5%
Glycero-phospholipids	LysoPC	18:1	C26H52NO7P	[M+FA-H]-	1.30	521.348	566.344	4	1.00E-04	-62.5%
Glycero-phospholipids	LysoPC	18:2	C26H50NO7P	[M+FA-H]-	1.05	519.333	564.330	1	3.18E-04	-78.2%
Glycero-phospholipids	LysoPC	20:3	C28H52NO7P	[M+FA-H]-	1.14	545.348	590.346	0	7.00E-04	-77.9%
Glycero-phospholipids	LysoPC	20:4	C28H50NO7P	[M+FA-H]-	1.01	543.333	588.331	0	2.62E-04	-73.1%
Glycero-phospholipids	PC	32:0	C40H80NO8P	[M+FA-H]-	7.70	733.562	778.560	0	2.58E-03	111.7%
Glycero-phospholipids	PC	32:1	C40H78NO8P	[M+FA-H]-	6.04	731.547	776.545	0	1.29E-04	62.5%
Glycero-phospholipids	PC	O-34:1/ P-34:0	C42H84NO7P	[M+FA-H]-	9.31	745.599	790.599	2	2.00E-03	84.3%
Glycero-phospholipids	PC	34:1	C42H82NO8P	[M+FA-H]-	7.94	759.578	804.578	2	8.21E-03	35.1%
Glycero-	PC	34:2	C43H82NO10P	[M+FA-H]-	6.42	757.562	803.568	0	1.29E-04	40.2%

phospholipids				isotope						
Glycero-phospholipids	PC	38:3	C46H86NO8P	[M+FA-H]-	9.13	811.609	856.607	0	2.51E-03	-30.7%
Glycero-phospholipids	PC	38:5	C46H82NO8P	[M+FA-H]-	6.27	807.578	852.576	0	1.29E-04	-28.8%
Glycero-phospholipids	PE	36:2	C41H78NO8P	[M-H]-	9.16	743.547	742.541	2	2.51E-03	54.4%
Glycero-phospholipids	PS	39:3	C45H82NO10P	[M+FA-H]-	7.94	827.568	872.567	1	4.42E-03	37.5%
Sphingolipids	SM	d38:1	C43H87N2O6P	[M+FA-H]-	10.15	758.630	803.630	2	3.46E-03	-37.6%
Sphingolipids	SM	d39:1	C44H89N2O6P	[M+FA-H]-	11.70	772.646	817.645	1	4.42E-03	-52.1%
Sphingolipids	SM	d40:1	C45H91N2O6P	[M+FA-H]-	12.90	786.662	831.661	1	3.46E-03	-48.6%
Sphingolipids	SM	d40:2	C45H89N2O6P	[M+FA-H]-	10.59	784.646	829.646	2	2.58E-03	-60.2%
Sphingolipids	SM	d41:1	C46H93N2O6P	[M+FA-H]-	13.22	800.677	845.675	0	2.00E-03	-48.2%
Sphingolipids	SM	d41:2	C46H91N2O6P	[M+FA-H]-	12.16	798.662	843.661	1	4.42E-03	-55.3%
Sphingolipids	SM	d42:1	C47H95N2O6P	[M+FA-H]-	13.49	814.693	859.689	2	3.92E-04	-52.5%

Molecular species that were either not detected or not significantly different in a particular electro-spray ionisation (ESI) mode were omitted from the table. Abbreviations: Cer, ceramide; CE; cholesterol ester; LysoPC, lysophosphatidylcholine; LysoPE, lysophosphatidylethanolamine; PC, phosphatidylcholine; PE, phosphatidylehtanolamine; PS, phosphatidylserine; RT, retention time; SM, sphingomyelin.
