# Supplementary material for: Discovery of Infection Associated Metabolic Markers in Human African Trypanosomiasis
Source: PLoS Negl Trop Dis. 2015 Oct 27;9(10):e0004200. doi: 10.1371/journal.pntd.0004200 (PMC4624234; doi:10.1371/journal.pntd.0004200)
Supplement: S5 Table — (PDF) [file pntd.0004200.s006.pdf]

S5 Table. Confusion matrices of HAT vs. control classification ability of UPLC-MS discriminatory markers

	Using all 37 Discriminatory Markers		Using Top 5 Discriminatory Markers	
	**Actual HAT Patients**	**Actual Controls**	**Actual HAT Patients**	**Actual Controls**
Predicted HAT Patients	15 (TP)	2 (FP)	15 (TP)	2 (FP)
Predicted Controls	1 (FN)	12 (TN)	1 (FN)	12 (TN)
	Sensitivity = TP/[TP+FN] = 93.75%	Specificity = TN/[TN+FP] = 85.71%	Sensitivity = TP/[TP+FN] = 93.75%	Specificity = TN/[TN+FP] = 85.71%
Upper 95% CI	100%*	100%*	100%*	100%*
Lower 95% CI	81.89%	67.38%	81.89%	67.38%

Classification based on O-PLS-DA models (for 16 Markers: R^2Y 0.780, Q^2Y 0.555; for top 5 markers: R^2Y 0.558, Q^2Y 0.505). Abbreviations as described for S3 Table.
