# Supplementary material for: Discovery of Infection Associated Metabolic Markers in Human African Trypanosomiasis
Source: PLoS Negl Trop Dis. 2015 Oct 27;9(10):e0004200. doi: 10.1371/journal.pntd.0004200 (PMC4624234; doi:10.1371/journal.pntd.0004200)
Supplement: S1 File — (DOCX) [file pntd.0004200.s007.docx]

**S1 File**

**Supplementary Protocols for Statistical Analyses**

Presented NMR results were based on ^1^H CPMG spectra, which allowed clearer distinction of metabolite (with small molecular weight) peaks from broad protein/lipid signals. NMR datasets were mean centred and scaled to unit variance, to give each variable (peak) equal weighting for analysis, followed by PCA, PLS-DA and O-PLS-DA. For lipid UPLC-MS data, median integral values for features were imported into SIMCA software and Pareto scaled. PCA analyses was performed on features from all test samples, QC’s, and blank controls to identify and remove contaminant/artefactual features, present in either only blanks or QC’s, as described by Vorkas et al, 2015 [1]. Experimental reproducibility of both ESI+ and ESI- runs were subsequently evaluated with PCA models, based on features from test samples and QC’s only, whereby tight grouping of QC samples indicate analytical stability suitable for metabolic profiling. Once this was confirmed, QC samples were excluded and final analyses were performed on the test samples only, using PCA, PLS-DA and O-PLS-DA.

All models were evaluated using R^2^X model parameter, which shows the fraction of variation in the x data matrix (spectral peak intensities) explained by the model; also known as model fit parameter. Quality of PLS-DA and O-PLS-DA models were further assessed by their R^2^Y values (which describes the fraction of variation in the sample classifier — HAT vs. control — y-matrix explained by the model) and Q^2^Y values (parameter for model predictive ability). The latter is automatically calculated using a 7-fold leave-one-out cross-validation of dataset in SIMCA. PLS-DA were further validated using permutation testing in SIMCA, whereby sample classifiers were scrambled 999 times and models re-calculated to establish the likelihood of achieving the same result by chance.

For UPLC-MS data, the variables responsible for the separation observed in the O-PLS-DA scores plots were visualised in corresponding S-plots, in which the integral of each metabolite feature is represented as a single co-ordinate [2]. The covariance of variables (p[1]) is shown along the x-axis, where variables furthest horizontally from the zero value display the highest contribution towards the model. The correlation coefficient of each feature with their predicted class is plotted along the y-axis (p(corr)[1]), representing the reliability of that feature (the most reliable being furthest vertically away from zero in the y-axis). Threshold criteria for initial selection of markers were variables with a correlation value p(corr)[1]>0.5 and covariance p[1]>0.05, to extract the most discriminatory and robust features.

Integrals of discriminatory variables (performed separately for NMR and UPLC-MS data) were re-imported into SIMCA as new loadings for O-PLS-DA models to evaluate the accuracy of the collection of these metabolites for biomarker potential. These models was performed twice: firstly, models were generated using integrals of all significantly discriminatory metabolites, and secondly using only integrals of the top 5 discriminating metabolites, defined as those which displayed the greatest % difference between patients and controls. Numbers of samples that were correctly assigned to either HAT or controls, according to the group separation along the first predictive component, were used to calculate combined sensitivity (true positives/[true positives + false negatives]) and specificity (true negatives/[true negatives + false positives]) of assessed discriminatory variables in patient/control classification. 95% binomial proportion confidence intervals (CI) were subsequently calculated for each measured sensitivity/specificity value.
